# Long-Term Hemodynamic Effects of Portocaval Shunt and Sugiura Procedure in Patients with Cirrhosis

**DOI:** 10.1155/1996/46476

**Published:** 1996

**Authors:** Corinne Vons, Antoine Hadengue, Claude Smadja, Dominique Franco, Didier Lebrec

**Affiliations:** 1 From the Groupe de Recherche sur la Chirurgie du Foie et de l'Hypertension Portale Hôpital Antoine Béclère Clamart and from Unité de Recherches de Physiopathologie Hépatique INSERM U 24, Hôpital Beaujon Clichy France; 2 Service de Chirurgie Hôpital Antoine Béclère 157 rue de la Porte de Trivaux Clamart cedex 92141 France

## Abstract

Systemic and splanchnic hemodynamics were studied before and six months after a portal systemic shunt (n=6) or a Sugiura procedure (n=9) in 15 patients with cirrhosis and a past history of variceal bleeding. Hepatic blood flow was estimated by hepatic extraction and clearance of continuous indocyanine green infusion. Azygos blood flow was measured with a continuous thermodilution catheter. After portocaval shunt, the cardiac index increased significantly from 4.0±1.4 to 5.4±0.8 l/min m2 (p<0.05), the hepatic venous pressure gradient and hepatic blood flow were significantly decreased from 21±3 to 13±5 mm Hg (p<0.05) and from 1.20±0.35 to 0.37±0.16 l/min (p<0.05) respectively. The decrease in azygos blood flow was not significant (0.51±0.31 vs 0.25±0.33 l/min; p=0.1). After Sugiura procedure, there was no significant change in cardiac index, hepatic venous pressure gradient, hepatic blood flow or azygos blood flow. This is the first study to show the long-term maintenance of splanchnic and systemic hemodynamics in patients with cirrhosis after Sugiura procedure. The absence of long-term hemodynamic alterations could explain the absence of encephalopathy after this procedure.


*HPB Surgery*, 1996, Vol.9, pp.209–213
Reprints available directly from the publisher
Photocopying permitted by license only

© 1996 OPA (Overseas Publishers Association)
Amsterdam B.V. Published in The Netherlands
by Harwood Academic Publishers GmbH
Printed in Malaysia

### Long-Term Hemodynamic Effects of Portocaval Shunt and Sugiura Procedure in Patients with Cirrhosis

CORINNE VONS*, ANTOINE HADENGUE, CLAUDE SMADJA, DOMINIQUE FRANCO and DIDIER LEBREC

From the Groupe de Recherche sur la Chirurgie du Foie et de l'Hypertension Portale, Hôpital Antoine Béclère, Clamart and from Unité de Recherches de Physiopathologie Hépatique, INSERM U 24, Hôpital Beaujon, Clichy, France

*(Received 29 June 1994)*

Systemic and splanchnic hemodynamics were studied before and six months after a portal systemic shunt ($n = 6$) or a Sugiura procedure ($n = 9$) in 15 patients with cirrhosis and a past history of variceal bleeding. Hepatic blood flow was estimated by hepatic extraction and clearance of continuous indocyanine green infusion. Azygos blood flow was measured with a continuous thermodilution catheter. After portocaval shunt, the cardiac index increased significantly from 4.0±1.4 to 5.4±0.8 l/min m2 ($p < 0.05$), the hepatic venous pressure gradient and hepatic blood flow were significantly decreased from 21±3 to 13±5 mm Hg ($p < 0.05$) and from 1.20±0.35 to 0.37±0.16 l/min ($p < 0.05$) respectively. The decrease in azygos blood flow was not significant (0.51±0.31 vs 0.25±0.33 l/min; $p = 0.1$). After Sugiura procedure, there was no significant change in cardiac index, hepatic venous pressure gradient, hepatic blood flow or azygos blood flow. This is the first study to show the long-term maintenance of splanchnic and systemic hemodynamics in patients with cirrhosis after Sugiura procedure. The absence of long-term hemodynamic alterations could explain the absence of encephalopathy after this procedure.

KEY WORDS: Portocaval shunt Sugiura long-term hemodynamic

### INTRODUCTION

Surgical prevention of recurrent variceal bleeding in patients with portal hypertension can be obtained by decreasing variceal pressure by portal systemic shunting or eradicating esophageal varices. In patients with cirrhosis, the hemodynamic changes resulting from portal blood diversion may increase the prevalence of hepatic encephalopathy and decrease survival rate[1,2]. The Sugiura procedure[3], including splenectomy, extensive esophago-gastric devascularization, and oesophageal transection should not decrease portal pressure and hepatic blood flow and should be therefore better tolerated. However it has been suggested that splenectomy may decrease portal blood flow and portal pressure. Finally the hemodynamic effects of these two procedures are not well known. The aim of this study was to compare changes in systemic and splanchnic hemodynamics six months after a Sugiura procedure or a portocaval shunt in 15 patients with cirrhosis, portal hypertension and a past history of variceal bleeding.

### PATIENTS AND METHODS

### *Patients*

Fifteen consecutive patients with alcoholic cirrhosis were included in a prospective randomized trial comparing surgical portocaval shunts to a Sugiura procedure in the prevention of recurrent variceal bleeding. Inclusion criteria were:

*Correspondence to*: DR. C. Vons, Service de Chirurgie, Hôpital Antoine Béclère, 157, rue de la Porte de Trivaux, 92141 Clamart cedex. Telephone number: (1) 45374347; Fax: (1) 45374978.

a) at least one episode of variceal bleeding confirmed by endoscopy; b) serum bilirubin < 50 µmol/l; c) prothrombin time > 40% of control; d) no biological or morphological signs of hepatocellular carcinoma; e) patent portal vein on superior mesenteric and celiac arteriograms. There were 10 males and five females. Mean age was 51±8 years. Six patients had a portocaval shunt and nine had a Sugiura procedure. Liver tests were performed before surgery. Patients were classified according to the Pugh Score[4]. There were no significant differences between the two groups (Table 1).

*Operative Procedure*

Patients were operated on after a mean interval of 8±2 weeks following variceal hemorrhage when they had fully recovered from bleeding.

Portocaval shunts were performed through a right subcostal incision. A side-to-side portocaval shunt was performed in five patients (mean decrease during surgery in portal pressure: 11±3 mm Hg) and a mesocaval shunt (Dacron, 18 mm in diameter) in one patient. Sugiura procedure was performed through a single left subcostal abdominal approach and included esophago-gastric devascularization, splenectomy and esophageal transection[5]. The lower third of the esophagus was devascularized from the left lower pulmonary vein to the diaphragm, requiring ligation of collateral veins joining peri-esophageal and intra-esophageal varices. The esophagus was divided and anastomosed at the esogastric junction with a mechanical stapler to interrupt the flow through the submucosal veins[6].

*Hemodynamic Investigations*

Hemodynamic measurements were performed as previously described[7], preoperatively, 10 to 21 days before surgery, and postoperatively six months after surgery. Briefly, in the morning after an overnight fast, patients were premedicated with 50 mg meperidine intramuscularly and placed on a padded fluoroscopy table. The right internal jugular vein was canulated with a 9F introducer sheath to allow insertion of different catheters. The hepatic venous pressure gradient, calculated as the difference between the wedged and free hepatic venous pressures, was measured by a precurved catheter (Cordis SA, Miami, FL). A Swan-Ganz catheter was used to measure pulmonary arterial and capillary wedged pressures and cardiac output. The systemic vascular resistance (SVR) was calculated by the following formula: SV = (mean arterial pressure-right atrial pressure) X 80/ cardiac output. Hepatic blood flow was estimated by hepatic extraction and clearance of continuous indocyanine green infusion (8). This value was rejected if extraction was 10% or less and if a steady-state concentration was not achieved. Azygos blood flow was measured with a continuous thermodilution catheter introduced into the arch of the azygos vein; the details of this procedure have been previously described[9]. Heart rate and mean arterial pressure were monitored in all patients. Informed witnessed consent was obtained from all patients.

*Statistical Analysis*

Results are expressed as mean ± SE. Results were analyzed by paired and unpaired student t test.

**Table 1** Liver biological tests before and 6 months after portocaval shunt and Sugiura procedure in cirrhotic patients.

| | *Portocaval shunt (6 patients)* | | *Sugiura procedure (9 patients)* | |
|---|---|---|---|---|
| | *Before* | *After* | *Before* | *After* |
| Serum billirubin (µ mol/l) | 22±5 | 42±12* | 23±7 | 17±5 |
| Serum albumin (g/l) | 34±9 | 30±4 | 36±4 | 41±4 |
| Prothrombin time (%of control) | 74±12 | 52±22* | 58±11 | 77±10 |
| Pugh Score A (Nb. of patients) | 4 | 2 | 7 | 9 |
| Pugh score B (Nb. of patients) | 2 | 4 | 2 | 0 |

*significantly different from before (p < 0.05).

### RESULTS

Six months after portocaval shunt, sonographic examination demonstrated a patent shunt in all six patients. An asymptomatic thrombosis of the portal vein was discovered in one patient after a Sugiura procedure. After portocaval shunt, esophageal varices disappeared in all patients. After the Sugiura procedure, three patients still had varices, which were small in two and large in one.

Results of liver tests six months after surgery are listed in Table 1. After Sugiura procedure, prothrombin time increased slightly, but the difference with the preoperative value was not significant. After portocaval shunt, serum bilirubin increased and prothrombin time decreased significantly ($p < 0.05$). One transient episode of encephalopathy was noted in one patient after a portocaval shunt. No encephalopathy occurred after the Sugiura procedure.

Pre and postoperative values of cardiac index, mean arterial pressure, heart rate and systemic vascular resistance are indicated in Table 2. In patients with a portocaval shunt, there was a significant ($p < 0.05$) increase in cardiac index (35%). None of the systemic hemodynamic values was significantly modified after the Sugiura procedure. The hepatic venous pressure gradient and hepatic blood flow decreased significantly by 26% and 50% respectively after portocaval systemic shunt. Azygos blood flow, measured in four patients after the surgical shunt, decreased in two patients but did not change in two others. There were no alterations of the splanchnic hemodynamic values after the Sugiura procedure (Table 3).

**Table 3** Splanchnic hemodynamics before and 6 months after portal systemic shunt or Sugiura procedure in cirrhotic patients.

| | *Portocaval shunt (6 patients)* | | *Sugiura procedure (9 patients)* | |
|---|---|---|---|---|
| | *Before* | *After* | *Before* | *After* |
| Wedge hepatic venous pressure (mm Hg) | 27.6±4.0 | 20.4±7.0 | 22.0±4.0 | 22.0±5.0 |
| Free hepatic venous pressure (mm Hg) | 6.6±7.0 | 7.4±4.0 | 7.8±4.0 | 6.0±4.0 |
| Hepatic venous pressure gradient (mm Hg) | 21.0±3.0 | 13.0±5.0 | 14.6±2.0 | 15.7±5.0 |
| Total hepatic blood flow (l/min) | 1.20±0.35 | 0.37±0.16* | 1.40±0.66 | 1.10±0.74 |
| Azygos blood flow (l/min) | 0.51±0.31 | 0.25±0.33** | 0.47±0.23 | 0.47±0.20 |

*significantly different from before ($p < 0.05$).
**$p < 0.1$

### DISCUSSION

The results of this study demonstrate that significant changes in splanchnic and systemic hemodynamics occur after portocaval shunt but not after Sugiura procedure in patients with cirrhosis. In the former group, hepatic bloodflow and pressures decreased and cardiac output increased. Six months after the Sugiura procedure there were no significant alterations of hepatic venous pressures, hepatic blood flow or cardiac index.

Alterations of splanchnic hemodynamics after portocaval shunt have previously been documented[10–13]. The results of this series confirm the reduction of hepatic venous pressures following portocaval shunt[13] and show that this reduction persisted for at least six months. Changes in azygos blood flow after portocaval shunt differ from one patient to another, but the decrease was not significant. Patterns of portosystemic collaterals varcies in patients with portal hypertension[14] and may explain these discrepancies. However, these results differ from a previous study where azygos blood flow was found to be significantly lower in five patients with cirrhosis after distal splenorenal shunt[15] than in five patients who did not undergo shunt surgery.

Discrepant findings have been obtained in previous reports concerning the influence of Sugiura procedure on free and wedged hepatic venous pressures[16,17]. In patients with hepatic schistosomiaous, it has been shown that portal pressure decreased following splenectomy associated with esophageal devascularization up to two weeks after the operation[16, 17]. In another study performed in patients with cirrhosis, the hepatic venous pressure gradient significantly decreased one month after the Sugiura procedure[18]. In the present study as in these two previous studies, portal pressure estimated by wedged hepatic venous pressure, did not change after the Sugiura procedure. However, in other studies also performed in patients with cirrhosis both during the operation and in early postoperative measurements, gastroesophageal devascularization suppressed the effect of splenectomy on hepatic venous pressure[19, 20]. Measurements of portal or hepatic blood flow after Sugiura procedure have provided different results. Portal blood flow, which has been measured

**Table 2** Systemic hemodynamics before and 6 months after portocaval shunt or Sugiura procedure in cirrhotic patients.

| | *Portocaval shunt (6 patients)* | | *Sugiura procedure (9 patients)* | |
|---|---|---|---|---|
| | *Before* | *After* | *Before* | *After* |
| Heart rate (beats/min) | 97±32 | 90±7 | 76±13 | 75±17 |
| Mean arterial pressure (mm Hg) | 89±19 | 92±7.8 | 89±12 | 96±12 |
| Cardiac index (l/min m2) | 4.0±1.4* | 5.4±0.8* | 4.0±1.0 | 3.8±0.6 |
| Systemic resistance (dyn /cm$^2$) | 976±407 | 735±108 | 1029±487 | 1143±364 |

*significantly different from before ($p < 0.05$).

both intraoperatively by the thermodilution technique and two weeks after the operation using a pulsed Doppler flowmeter[19, 20], was found to be decreased. However, Kawazaki et al showed that hepatic blood flow, the sum of portal vein and hepatic arterial blood flows, measured by the galactose clearance technique, was maintained during the early postoperative follow-up[16]. The results of the present study confirm the maintenance of hepatic blood flow using hepatic extraction and clearance of indocyanine green. Although the decrease in portal blood flow and the maintenance of hepatic blood flow may seem contradictory, these results are not suprising, since the reduction of portal blood flow is probably compensated by an increase of hepatic arterial blood flow.

Finally, the results of this series of patients demonstrated that splenectomy associated with esophagogastric devascularization and esophageal transection has no deleterious influence on splanchnic hemodynamics. The absence of alterations in splanchnic hemodynamics after the Sugiura procedure was maintained for a long-term period.

After the Sugiura procedure, azygos blood flow alterations differed from one patient to another but the mean value did not change. It has been previously suggested that splenectomy decreased the azygos blood flow in patients with cirrhosis[21]. Kokudo *et al.*[18] demonstrated a significant decrease in azygos blood flow one month after the Sugiura procedure. However results of this study suggest that azygos blood flow is maintained for at least six months. Late preservation of azygos blood flow in some patients, despite esophageal devascularization, suggests that most azygos blood flow comes from periesophageal collateral circulation which is not interrupted by the Sugiura procedure. However, persistance of elevated azygos blood flow might, at least, favor recanalization of esophageal varices.

Alterations of systemic hemodynamics have been previously demonstrated after portocavalshunt[14] but not after the Sugiura procedure. Indeed, the marked increase in cardiac output after portocaval shunt, resulting from the increase in venous return, has been previously demonstrated[11]. In contrast with portocaval shunt, cardiac output and vascular systemic resistance did not significantly change after the Sugiura procedure. This result is probably because the Sugiura procedure did not alter splanchnic hemodynamics.

Finally, in this series, associations between hemodynamic and liver tests alterations were observed. After portocaval shunt, serum bilirubin increased and serum albumin and prothrombin time decreased, while after the Sugiura procedure there were no liver test modifications. The absence of splanchnic hemodynamic alterations after the Sugiura procedure migh explain this, suggesting that this procedure might be better tolerated than portocaval shunt in patients with cirrhosis.

### REFERENCES

1. Rypins EB, Sarfeh IJ. (19[illegible] Influence of portal hemodynamics on long-term survival [illegible] alcoholic cirrhotic patients after small-diameter portacaval H grafts. *Am J Surg* , **155**: 152–7.
2. Henderson JM, Millikan WJ, Wright L, Kutner MH, Warren WD. (1982) Quantitative estimation of metabolic and hemodynamic hepatic function: the effects of shunt surgery. *Surg Gastroenterol*, **1**: 77–85.
3. Sugiura M, Futagawa S. (1973) A new technique for treating esophageal varices. *J Thorac Cardiovasc Surg*, **66**: 677–85.
4. Pugh RNH, Murray-Lyon IM, Dawson JL, Pietroni MC,Williams R. (1973) Transection of the esophagus for bleeding esophageal varices. *Br J Surg*, **60**: 646–9.
5. Umeyama K, Yoshikawa K, Tamashita T, Todo T, Satake K. (1983) Transabdomina esophageal transection for esophageal varices: experience in 101 patients. *Br J Surg*, **70**: 419–22.
6. Ginsberg RJ, Waters PF, Zeldin RA, Spratt EH, Shandling B, Stone RM, Strasberg S. (1982) A modified Sugiura procedure. *Ann Thorac Surg*, **34**: 258–63.
7. Vonc C, Hadengue A, Lee SS, Smadja C, Franco D, Lebrec D. (1991) Splanchnic and systemic hemodynamics in cirrhotic patients with refractory ascites. Effect of peritoneovenous shunting. *HPB Surgery*, **3**: 259–69.
8. Caesar J, Shaldon S, Chiandusi L, Guevara L, Sherlock S. (1961) The use of indocyanine green in the measurements of hepatic blood flow and as a test of hepatic function. *Clin Sci*, **21**: 43–57.
9. Schrier RW, Arroyo V, Bernardi M, Epstein M, Henriksen JH, Rodes J. (1988) Peripheral arterial vasodilatation hypothesis: a proposal for the initiation of renal sodium and water retention in cirrhosis. *Hepatology*, **8**: 1151–7.
10. Steegmuller KW, Marklin HM, Hollis HW. (1984) Intraoperative hemodynamic investigations during portacaval shunt. *Arch Surg*, **119**: 269–73.
11. Reichle FA, Owen OE. (1979)Hemodynamic patterns in human hepatic cirrhosis. A prospective randomized study of the hemodynamic sequeale of distal splenorenal (Warren) and mesocaval shunts. *Ann Surg*, **190**: 523–34.
12. Rikkers LF, Miller FJ, Christian P. (1981) Effect of portasystemic shunt operations on hepatic portal perfusion. *Am J Surg*, **141**: 169–74.
13. Reynolds TB. (1974) The role of hemodynamic measurements in portosystemic shunt surgery. *Arch Surg*, **108**: 176–81.
14. Rousselot LM, Burchell AR. (1975) Splenic and arterial portography and hemodynamics in portal hypertension. In: Schiff L ed. Disease of the Liver. Philadelphia: *JB Lippincott*, 368–423.
15. Bosch J and Groszmann R.J. (1984) Measurement of azygos venous blood flow by a continuous thermal dilution technique: An index of blood flow through gastroesophageal collaterals in cirrhosis. *Hepatology*, **4**: 424–9.
16. Kawasaki S, Kidokoro A, Sugiura M, Sanjo K, Idezuki Y. (1987) Effects of non shunting operations on portal venous pressure and hepatic blood flow. *Am J Surg*, **153**: 295–9.

17. Karara K, EL-Gendi MA, Gertsch PH, Mosimann R. (1987) Portal pressure measurements before and after Hassab's decongestion operation. A preliminary report. *Int Surg*, **72**: 141–3.
18. Kokudo N, Kawasaki S, Ohashi K, Sakamoto H, Koyama H, Sanjo K, Idezuki Y. (1989) Effects of non-shunting operations on azygos venous blood flow in cirrhotic patients. *Gut* **30**: 1396–400.
19. Saito M, Ohnishi K, Tanaka H, Sato S, Okuda K, Hirashima T, Hara T. (1987) Effects of esophageal transection combined with splenectomy on portal hemodynamics. *Am J Gastroenterol*, **82**: 16–9.
20. Takenaka H, Nakao K, Miyata M, Nakahara M, Nagaoka M, Hashimoto T, Kawashima Y. (1990) Hemodynamic study after devascularization procedure in patients with esophageal varices. *Surgery*, **107**: 55–62.
21. Hadengue A, Lee SS, Moreau R, Lebrec D. (1987) Oxygen and bile acid content in the azygos blood. Clues to the azygos derivation in patients with portal hypertension. *J Hepatol*, **5**: 98–101.

